# Case report: Daratumumab treatment in pre-transplant alloimmunization and severe hemolytic anemia

**DOI:** 10.3389/fimmu.2022.1055473

**Published:** 2022-11-29

**Authors:** Maria A. Pereda, Smitha Hosahalli Vasanna, Neha J. Desai, Victoria Deng, Amma Owusu-Ansah, Mari H. Dallas, Irina Pateva, Jignesh Dalal

**Affiliations:** ^1^ Department of Pediatrics, Division of Pediatric Hematology Oncology, University Hospitals Rainbow Babies & Children’s Hospital, Cleveland, OH, United States; ^2^ School of Medicine, Case Western Reserve University, Cleveland, OH, United States

**Keywords:** daratumumab, CD38 monoclonal antibody, hematopoietic stem cell transplantation, pediatrics, non malignant

## Abstract

Daratumumab, a CD38 monoclonal antibody that has been FDA-approved to treat multiple myeloma, has acquired popularity and is used off-label for both auto- and alloantibody mediated disorders, particularly in refractory/resistant circumstances. Much of the published data for its use in pediatric blood disorders has been in post-transplant autoimmune cytopenias. Here we describe three patients in whom daratumumab was used outside of post-transplant autoimmune cytopenias, highlighting further potential uses of this medication.

## Introduction

Daratumumab (DARA) is a human IgG1k monoclonal antibody that specifically targets CD38, a protein expressed on plasma blasts and plasma cells (PCs). CD38 expression is highest in normal plasma cells and multiple myeloma cells (1). The US Food and Drug Administration (FDA) approved DARA in 2015 for the treatment of relapsed/refractory multiple myeloma (MM). Off-label usage of this medication in numerous antibody-mediated auto/alloimmune diseases refractory to conventional therapies has increased in recent years. Conventional treatment for antibody-mediated diseases includes steroids, intravenous immunoglobulin (IVIG), and B-cell depleting therapies like anti-CD20 monoclonal antibody (rituximab) (2). Even though rituximab is regarded as a major breakthrough in the treatment of autoimmune diseases, a subset of patients fail to respond to it. While a complete response to rituximab is associated with short-lived PCs, a primary non-response is related to auto-reactive long-lived PCs either in the bone marrow and/or in the inflamed tissues (3). Furthermore, a paradoxical increase in plasma cells has been described in the context of B-cell depletion leading to chronicity of autoimmune diseases (3). Hence, plasma cell depletion with DARA has emerged as an attractive therapeutic strategy for patients who are resistant/refractory to rituximab. Also, a newer concept of combining rituximab with DARA to target both B-cells and PCs to eliminate the sources of antibody production in refractory patients has been tried anecdotally.

In published literature so far, DARA has been used successfully for the treatment of post-allogenic hematopoietic stem cell transplantation (HSCT) associated autoimmune cytopenias (AIC) and pure red cell aplasia, refractory autoimmune hemolytic anemias (AIHA) and autoimmune thrombocytopenia (AIT), antibody-mediated rejection of transplanted kidney, antiphospholipid syndrome, systemic lupus erythematosus, and proliferative glomerulonephritis (4–14).

Daratumumab’s mechanisms of action include complement-dependent cytotoxicity, antibody-dependent cellular cytotoxicity, antibody-dependent cellular phagocytosis, apoptosis, enzymatic activity regulation, and immunomodulatory effects. DARA has modest toxicity profile, with the most common side effects described being infusion-related anaphylactic reactions and increased risk of infection (15, 16). In MM clinical trials, using 16 mg/kg of intravenous DARA weekly, 37% patients experienced infusion-related reactions in the first week, with rates decreasing with successive infusions (17–19). On immune cells such as natural killer cells, T and B cells, and erythrocytes, low surface CD38 expression is observed. Daratumumab’s interaction with these cells can lead to unintended consequences such as interference with cross-matching of blood products, impairment in polyclonal immunoglobulin production, and potential immunomodulation *via* regulatory T-cell elimination (20–22).

There is limited data on off-label use of DARA in antibody-mediated diseases in pediatric patients outside of post-transplant AIC.

## Methods

We report a series of three pediatric patients who received DARA in a variety of novel alloimmunization settings. The first patient, with sickle cell disease, had severe red cell alloimmunization. In the second patient with sickle cell disease, DARA was used as part of a transplant conditioning regimen. The third patient, with severe aplastic anemia, had multiple HLA antibodies directed against the maternal haploidentical donor, likely contributing to transfusion refractoriness.

## Clinical features and course

### Case 1

A 17-year-old female with sickle cell disease type SS whose disease had progressively worsened over the past two years and was hospitalized numerous times for vaso-occlusive episodes (VOE), acute chest syndrome (ACS), and delayed hemolytic transfusion reactions (DHTR). Multiple red cell allo-antibodies were detected as shown in **Supplementary Material Table A**. Our patient experienced severe anemia during each admission for pain crisis, requiring red blood cell transfusions. With every blood transfusion, steroids were given to prevent DHTR, which resulted in rebound pain crises. Her transfusion threshold was reduced to 5.5g/dL due to a history of allo-antibodies and difficulty procuring matched red blood cells. She received three distinct types of disease-modifying treatments. When her condition worsened while on hydroxyurea, crizanlizumab was introduced, but without much effect. Voxeletor was initiated to increase hemoglobin levels and reduce the need for blood transfusions. She received four doses of rituximab 375 mg/m^2^ to lessen the burden of red cell alloantibodies.

Due to the severe disease phenotype, curative options like HSCT and gene therapies were taken under consideration. Due to lack of a matched sibling donor and the patient not qualifying for gene therapy due to her young age, alternative donor transplant options were investigated. Given a prior sub-optimal response to rituximab, the decision to use DARA was made to eradicate red cell allo-antibodies.

**Figure 1:**
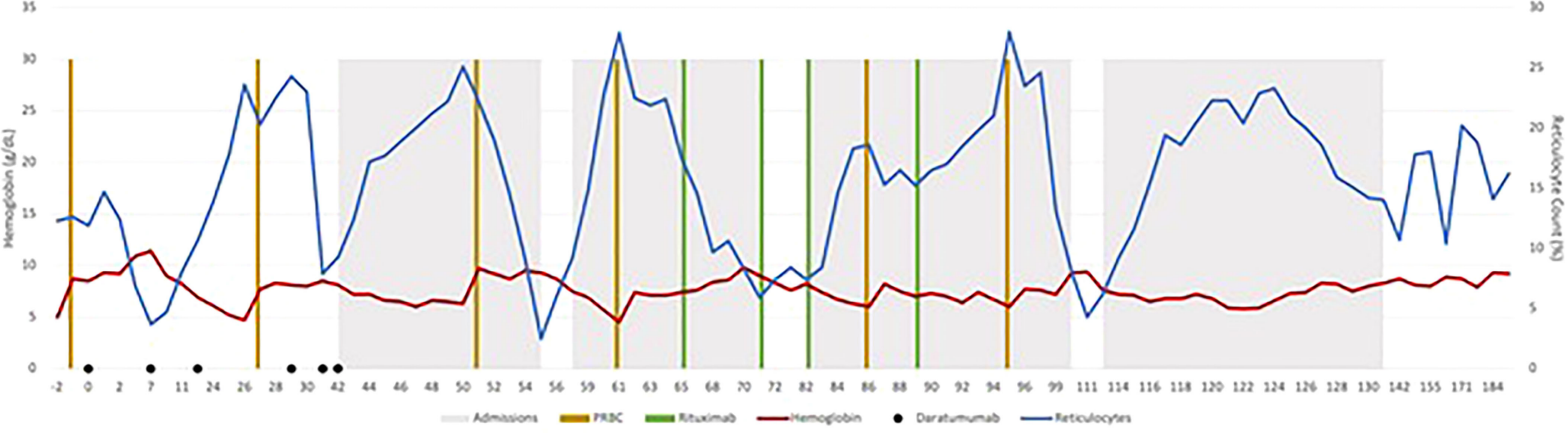
Effect of various treatments on the hemoglobin and reticulocyte count for patient 1.

Before starting DARA, our patient’s antibody profile was positive for anti little e, Anti-E, Anti-Fy, Anti-C and Anti-D (**Supplementary Material Table A**). Patient received weekly DARA for a total of 6 doses (16mg/kg per week), with the last dose given on day 39. She required intermittent PRBC transfusion until 93 days after the first dose of DARA. Her hemoglobin remains stable (7.2 – 9.5g/dL) since (**Figure 1**). As early as 10 days after treatment, our patient’s antibody profile improved with Anti-E and Anti-Fy becoming undetectable. Three months after completing treatment, all her previously positive alloantibodies became negative. She did not experience any side effects that were attributable to DARA. Previously described red cell allo-antibodies continue to remain negative one year post DARA.

### Case 2

A 14-year-old female with sickle cell disease, type SS, underwent a matched related donor HSCT in from her HLA-identical brother. She had developed anti-Fya, anti-E, anti-C, anti-Jka, and HTLA-like antibodies because of the several blood transfusions she had received prior to her HSCT (high-titer, low avidity). Due to an episode of severe delayed hemolytic transfusion reaction in October 2017 (nine months before first transplant), she received IVIG 1 gm/kg x 3 days and methylprednisone 2 mg/kg/day for a week followed by a prolonged steroid wean over 4 weeks and rituximab (375 mg/m2/dose) x 4 doses. Due to persistence of Anti-Fya antibodies were discovered through antibody screening done before transplant Day (T) =0 and hence received intravenous immunoglobulin (IVIG) 1 gm/kg x 1 dose two weeks prior to HSCT. During conditioning with Campath, fludarabine, and melphalan, she experienced a DHTR with a hemoglobin nadir of 3.8 g/dL. The donor was anti-Fya negative, so a red blood cell exchange transfusion was done. On T+18, engraftment failure with autologous recovery was identified as chimerism showed 98% recipient DNA.

At her first outpatient visit (T+38), her hemoglobin was 5.3 g/dL, and ranged between 6 g/dL and 9.7 g/dL over the course of the next seven months with transfusion support. She received four cycles of bortezomib 1.3 mg/m2/dose, intravenously every 72 hrs x 4 doses (T+7 months) without any clinical or laboratory improvement. One month after her last bortezomib dose, an extended antibody screening revealed persistently anti-Fya and anti-E (poly-ethylene glycol reacting) antibody titers. Anti-Fya titer was 1 and anti-E was not tested. Then, between T+8 and T+9 months, she received methylprednisone 2 mg/kg/day x 5 days, and IVIG 1 gm/kg x 1 dose, and rituximab 375 mg/m2/dose weekly x 2 doses due to experiencing delayed hemolytic transfusion reaction. Repeat antibody testing remained unchanged. One year after her initial HSCT, she received a second matched related donor HSCT from her HLA-identical brother. Patient demonstrated anti-Fya antibodies prior to the second HSCT. The conditioning regimen used was ATG (T-12 to T-10), cyclophosphamide (T-9 to T-8) and targeted busulfan (T-5 to T-2). Additionally, as part of the conditioning regimen our patient had plasmapheresis performed on T-7 and a single dose of DARA (16mg/kg) given on T-6. Our patient experienced a severe delayed hemolytic transfusion reaction after receiving a unit of packed red blood cell, and hence DARA was added to the preparative regimen. Exchange transfusion was performed on T=0. She successfully engrafted on T+23 and achieved B-cell immune reconstitution with normal IgG and IgM on T+90. Her antibody screening obtained at T+1 year showed no allo- or auto-antibodies. Patient has remained transfusion free since engraftment with normal hemoglobin range of 12 g/dL to 14.6 g/dL. Peripheral blood chimerism is added in the (**Supplementary Material Table B**). No side effects attributable to DARA was experienced by her.

### Case 3

A previously healthy 19-year-old, African American female presented with severe pancytopenia in March 2021. Hemoglobin was 5.5 gm/dL, white blood cell count was 0.5 x 10^9^E/L, absolute neutrophil count was 0.02 x 10^9^E/L, and platelets were 1 x 10^9^E/L at the time of diagnosis. A bone marrow biopsy revealed notably hypocellular marrow with < 5% cellularity, without leukemic blasts or aberrant cytogenetics. Extensive work-up to rule out inherited causes of bone marrow failure was negative; hence she was diagnosed with idiopathic severe aplastic anemia (iSAA). She demonstrated severe platelet refractoriness up-front requiring frequent platelet transfusions. Work-up for platelet refractoriness demonstrated low corrected count increment (CCI) indicating immune-mediated platelet destruction. A high antibody titer for both class I and class II human leukocyte antigen (HLA) as well as anti-platelet antibodies were identified on day 7 after initial presentation (**Supplementary Material Table C**). She demonstrated 63 different HLA antibodies from both classes, with a strong mean florescence intensity (MFI) of >10,000 for multiple HLA antibodies.

**Figure 2:**
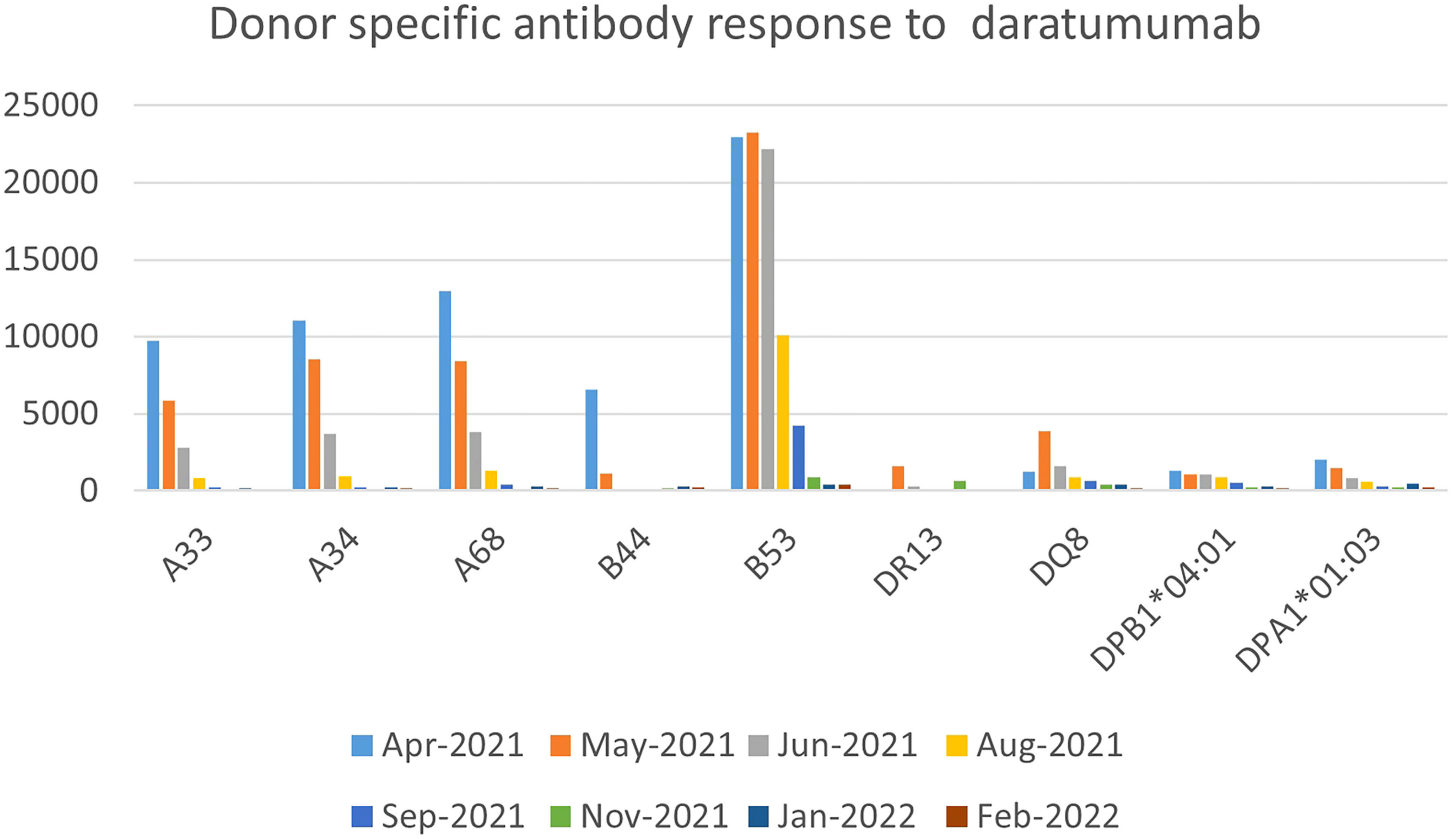
Donor specific antibody response to daratumumab.

She had received a total of 12 units of blood products including packed red cells and platelet, up until that point (five days since diagnosis of iSAA). She denied past medical history of blood transfusion or organ transplant, but obstetric history was significant for a miscarriage a year prior to presentation. In the absence of a suitable matched sibling donor or matched unrelated donor, she received immunosuppressive therapy (IST) with horse anti-thymocyte globulin and cyclosporine along with Eltrombopag. To address her severe antibody mediated platelet refractoriness, patient received a dose of IVIG 1 gm/kg based on ideal body weight and 2 doses of rituximab 375 mg/m^2^. Despite depletion of CD20 B-cells post-rituximab, she had no significant reduction in antibody burden. After 16 weeks of IST, our patient showed improved hemoglobin and absolute neutrophil count but continued needing platelet transfusions on a weekly basis. Due to failure of IST, work up for haploidentical HSCT was done which showed presence of donor specific antibodies (DSA), for which she received weekly daratumumab infusions (16 mg/kg/dose) for 5 weeks with a significant reduction in DSA and was able to receive a HSCT from a previously unsuitable haplo-identical donor (**Figure 2**). No side effects attributable to DARA was experienced by her.

## Discussion

The first 2 patients described in this case series developed alloimmunization secondary to frequent blood transfusions due to sickle cell disease. The etiology of anti-HLA antibodies for the third patient is suspected to be a previous miscarriage and autoimmunity.

Between 4.4% and 76% of sickle cell patients develop red blood cell (RBC) alloimmunization. RBC alloimmunization is not only a significant obstacle for transfusing patients with sickle cell disease due to the risk of severe hemolytic transfusion reactions, but also impacts transplant outcomes due to HLA alloimmunization (23). In a study by Nickel et al., HLA alloimmunization did not correspond with neutrophil engraftment, donor chimerism, or graft rejection in sickle cell patients, but RBC alloimmunization correlated with a decline in donor T cell chimerism at one year (24). Presence of donor specific HLA antibodies (DSA) indicates a 10 fold increase of primary graft failure and graft rejection, especially when MFI is > 10,000 (25, 26). Hence complete elimination/reduction of these RBC/HLA antibodies are crucial for successful transplant outcomes.

Multimodal therapies used to eliminate these antibodies includes strategies to remove antibody (therapeutic plasma exchange), reduce antibody production (steroids, rituximab, bortezomib, or immunosuppressive therapies), and inhibit complement cascade (IVIG). Patients failing multiple therapies have been postulated to harbor memory plasma cells leading to continued antibody production despite B cell depletion. In such refractory cases, DARA has shown to have promising results (27).

DARA has been reported to be effective against post-HSCT AIHA, post-HSCT autoimmune-like hepatitis, and delayed RBC engraftment in pediatric patients. There is only one report found in the pediatric literature that describes the successful use of single-dose DARA before HSCT to treat AIHA (9). A second patient with CGD who developed AIT after HSCT with graft failure did not respond to one dose of DARA and underwent a second HSCT 9 days after single-dose DARA (**Table 1**).

**Table 1:** Summary of published literature in the last 3 years using DARA in antibody mediated disorders in pre and post-transplant setting.

Authors	Primary disease/Timing of use of DARA with respect to HSCT	Reason for Daratumumab use	Failed prior line of treatments	Number of DARA doses	Outcomes
P. Khandelwal et al (28) BJH, 2021 (n=9)	Pre-Transplant (4): *IPEX syndrome*, *BCL11B def, WAS, BMF* Post-Transplant (5): *XIAP def, CTLA4 def, CGD, Griscelli syndrome*	AIHA (5), Autoimmune neutropenia (1), AIT (2), all (1)	IVIG, Corticosteroids, Rituximab, Abatacept, Bortezomib, plasmapheresis	1-5 doses	6 patients responded to Daratumumab at a median of 31 days from 1^st^ dose. 2 of the 6 patients had AIHA relapse. *First, 117 days after CR, 2^nd^ 380 days after CR.*
C. Schuetz et al. (5) Blood advances, 2021 (n=3)	Post-transplant: *B-ALL, WAS, DNA ligase IV deficiency*	AIHA	Corticosteroids, Mycophenolate Mofetil (MMF), sirolimus, rituximab, Cyclophosphamide, eculizumab, ibrutinib, plasmapheresis (n=1)	4 – 11 doses	2 patients achieved CR. One patient with transient response at 8 months died.
R. Epperly et al. (29) PBC, 2021 (n=1)	Post-transplant: *Ph B-ALL*	Autoimmune-like hepatitis	Corticosteroids	5 doses	CR and off corticosteroids
J.Koo et al. (2) PBC, 2020 (n=3)	Post- transplant: *Unknown*	AIHA	MMF, Ofatumumab, sirolimus, Rituximab, methylprednisolone	Unknown	Two of the three patients achieved CR and were off steroids.
E. Even-Or et al. (7) PBC, 2019 (n=1)	Post- transplant: *PS45 protein def*	AIHA	Prednisone, Rituximab, Abatacept, Bortezomib	6 doses	CR and off corticosteroids.
L. Cooling et al. (9) Transfusion, 2019 (n=1)	Pre-transplant: *BRAF V600E + LCH*	Warm antibody AIHA	Trametinib, prednisone, IVIG, Rituximab	1 dose	Improved and underwent HSCT after single dose.

IPEX syndrome, Immune dysregulation, polyendocrinopathy, enteropathy, X-linked syndrome; WAS, Wiskott Aldrich syndrome; BMF, bone marrow failure; XIAP, X-Linked Inhibitor of Apoptosis; CGD, chronic granulomatous disease; CR, complete remission; Ph B-ALL, Philadelphia positive B-Acute Lymphoblastic Leukemia; LCH, Langerhan’s cell histiocytosis.

In a recent case series of post-transplant AICs, only 25% of patients responded to first-line treatment with rituximab and steroids. Furthermore, increased rates of disease relapse and overall survival have been reported when DSA are present in patients who received an umbilical cord transplant and half of the patients of a different cohort who underwent successful desensitization relapsed despite successful primary engraftment (30–33). The regimen used in the latter cohort included plasmapheresis and rituximab. Other regimens using bortezomib and IVIG did not provide benefit. Our third patient was successfully transplanted with a haplo-identical donor for which she had DSA before treatment with DARA. Decreased MFI was evident as early as 1 month after her first dose of DARA and she achieved levels <500 MFI for all antibodies 2 months after completing all doses of DARA. There has been a prior report of the successful use of DARA for the treatment of therapy-refractory antibody-mediated rejection of a kidney transplant (34).

In pediatric patients with nephrotic syndrome, DARA has been explored in combination with obinutuzumab, a second-generation anti-CD20 monoclonal antibody with more powerful antibody-dependent cytotoxicity that can overcome low affinity of Fc-Gamma receptor 3A polymorphisms. In one case series, 9 of 14 patients remained in remission after a median follow-up of 22.5 months after first dose of obinutuzumab (35).

CD38 is a type II membrane glycoprotein that plays a role in adhesion, migration, signal transduction and generation of nucleotide metabolites. The therapeutic benefits of targeting CD38 have been explored in various clinical trials for MM. Hogan et al. (phase 2 DELPHINUS study - oral abstract) and Cerrano et al. recently published early results of the addition of DARA to re-induction therapy for relapsed/refractory T-ALL and lymphoblastic lymphoma showing improved response rates with manageable adverse events (36). In pediatrics, there is an ongoing clinical trial investigating the role of DARA in addition to standard chemotherapy for relapsed/refractory T or B cell ALL (NCT03384654). In addition, CD38 is an integral membrane protein that is expressed not only on plasma cell surface but also weakly on RBC surface, resulting in false positive antibody screens. A pan-agglutinin-like activity caused by anti-CD38 reactivity is usually reported in patients who receive DARA, which was easily distinguished from other reactive antibodies in our patients. This suggests that the use of DARA in AIHA should not be prevented by cross-reactivity in indirect antiglobulin tests. In the MM trials, the effects of DARA, whose half-life is 18 days+/- 9 days were observed as early as 1 month after administration. The standard dose of 16 mg/kg weekly for 6 to 9 weeks has been extrapolated from MM studies, although pediatric patients as well as those with nonmalignant disorders may respond to lower and fewer doses of DARA.

In general, DARA was very well tolerated, and side effects such as upper respiratory, gastrointestinal problems and infusion reactions as previously described were not reported in our patients. Patients who receive DARA should be screened for hepatitis B, as it is known to reactivate the virus. Our patients were managed as if immunocompromised and received *Pneumocystis jirovecii* prophylaxis based on prior studies showing the infection rate to be as high as 49% for MM patients on DARA (37). Prolonged neutropenia (~50 days) as a side effect of DARA monotherapy has been described in an elderly MM patient. Neutropenia was reported in phase 1-2 MM clinical studies in 5-12% of patients receiving DARA monotherapy, however the prevalence was higher (28–48%) when DARA triple treatment was used. Dose reduction/delaying administration of DARA is recommended with grade 3/4 hematological toxicities, and interruption when neutropenia (ANC 0.5 x 109/L) or thrombocytopenia (50 x 109/L) develops. However, when DARA is used for treatment of AIC, baseline count prior to initiation of DARA and clinical condition of the patient should be strongly taken into consideration before drug interruption. Hypogammaglobulinemia (IgG < 600) with risk of infection is a potential complication for patients on DARA. Paul et al. reported almost doubled incidence of hypogammaglobulinemia in MM patients with the use of DARA (38). Though two of our patients required IVIG replacement sporadically, none of them required periodic administration.

Multiple therapies were being concurrently used in our patients in order to reduce the antibody burden, making it challenging to attribute response just to DARA alone, which is one of the limitation of the study. In summary, we report 3 heterogenous clinical scenarios in which DARA provided resolution of antibodies against RBC minor blood group and HLA that were refractory to multiple therapies (**Table 2**). This improved not only the patients’ quality of life but allowed us to proceed with curative treatments such as allogeneic HSCT. Our case series highlights the use of daratumumab as a single agent to treat alloimmunization and DSA in patients needing a HSCT. We suggest that daratumumab could be used as part of a conditioning regimen in the presence of RBC alloimmunization.

**Table 2:** Summary of our patients with indications for Daratumumab and outcomes.

Patient No.	Primary disease	Reason for Daratumumab use pre-HSCT	Failed prior line of treatments	Number of Daratumumab doses	Outcomes
Patient 1	Sickle cell disease with red cell alloimmunization	Reduce alloantibody burden prior to transplant	Steroids, Rituximab	6 doses (16 mg/kg/dose per week)	Complete elimination of red cell alloantibodies by 3 months post daratumumab
Patient 2	Sickle cell disease with red cell alloimmunization and primary HSCT graft failure	Daratumumab used as a part of conditioning regimen prior to second HSCT	Steroids, IVIG, Rituximab, Bortezomib, Plasmapheresis	1 dose on T-6 of second HSCT	Successfully engrafted with second transplant. Antibody screen negative at T+1 year post second HSCT
Patient 3	Aplastic anemia with severe platelet refractoriness due to multiple high MFI titer HLA antibodies	To reduce/eliminate donor specific HLA antibodies	IVIG, Rituximab x 2 doses	5 doses	Significant reduction in the antibody burden. Doing well at T+172 days from HSCT

## Data availability statement

The datasets for this article are not publicly available due to concerns regarding participant/patient anonymity. Requests to access the datasets should be directed to jignesh.dalal@uhhospitals.org.

## Ethics statement

Written informed consent was obtained from all the involved participants and/or their legal guardians for the publication of any potentially identifiable data included in this article.

## Author contributions

MP formulated the idea. MP, SH-V, ND and VD were part of the writing of the manuscript, literature search. JD, IP, MD, AO-A helped growing the idea, reviewing the manuscript, provide excellent feedback to improve the manuscript content and helped further writing and with literature additions. All authors contributed to the article and approved the submitted version.

## Conflict of interest

The authors declare that the research was conducted in the absence of any commercial or financial relationships that could be construed as a potential conflict of interest.

## Publisher’s note

All claims expressed in this article are solely those of the authors and do not necessarily represent those of their affiliated organizations, or those of the publisher, the editors and the reviewers. Any product that may be evaluated in this article, or claim that may be made by its manufacturer, is not guaranteed or endorsed by the publisher.

## References

[1] Morandi F Horenstein AL Costa F Giuliani N Pistoia V Malavasi F. CD38: A target for immunotherapeutic approaches in multiple myeloma. Front Immunol (2018) 9:2722(NOV). doi: 10.3389/FIMMU.2018.02722 30546360 PMC6279879

[2] Koo J Giller RH Quinones R McKinney CM Verneris MR Knight-Perry J. Autoimmune cytopenias following allogeneic hematopoietic stem cell transplant in pediatric patients: Response to therapy and late effects. Pediatr Blood Cancer (2020) 67(9):e28591. doi: 10.1002/PBC.28591 32658382

[3] Crickx E Weill JC Reynaud CA Mahévas M. Anti-CD20-mediated b-cell depletion in autoimmune diseases: successes, failures and future perspectives. Kidney Int (2020) 97(5):885–93. doi: 10.1016/J.KINT.2019.12.025 32229095

[4] Driouk L Schmitt R Peters A Heine S Josef Girschick H Strahm B. Daratumumab therapy for post-HSCT immune-mediated cytopenia: experiences from two pediatric cases and review of literature. Mol Cell Pediatr (2021) 8(1):5. doi: 10.1186/S40348-021-00114-Y 33914175 PMC8085143

[5] Schuetz C Hoenig M Moshous D Weinstock C Castelle M Bendavid M. Daratumumab in life-threatening autoimmune hemolytic anemia following hematopoietic stem cell transplantation. Blood Adv (2018) 2(19):2550–3. doi: 10.1182/BLOODADVANCES.2018020883 PMC6177653 30291113

[6] Blennerhassett R Sudini L Gottlieb D Bhattacharyya A. Post-allogeneic transplant Evans syndrome successfully treated with daratumumab. Br J Haematol (2019) 187(2):e48–51. doi: 10.1111/bjh.16171 31441030

[7] Even-Or E Naser Eddin A Shadur B Dinur Schejter Y Najajreh M Zelig O. Successful treatment with daratumumab for post-HSCT refractory hemolytic anemia. Pediatr Blood Cancer (2020) 67(1):e28010. doi: 10.1002/PBC.28010 31544339

[8] Jain A Gupta DK. Daratumumab for refractory warm autoimmune hemolytic anemia. Ann Hematol (2021) 100(5):1351–3. doi: 10.1007/S00277-020-04063-W 32405694

[9] Cooling L Hugan S. Daratumumab in combination with standard treatment for autoimmune hemolytic anemia in a pediatric patient. Transfusion (Paris) (2019) 59(12):3801–2. doi: 10.1111/TRF.15539 31802512

[10] Crickx E Audia S Robbins A Boutboul D Comont T Cheminant M. Daratumumab, an original approach for treating multi-refractory autoimmune cytopenia. Haematologica (2021) 106(12):3198. doi: 10.3324/HAEMATOL.2021.279232 34348453 PMC8634173

[11] Kwun J Matignon M Manook M Guendouz S Audard V Kheav D. Daratumumab in sensitized kidney transplantation: Potentials and limitations of experimental and clinical use. J Am Soc Nephrol (2019) 30(7):1206–19. doi: 10.1681/ASN.2018121254/-/DCSUPPLEMENTAL PMC6622431 31227636

[12] Ostendorf L Burns M Durek P Anne Heinz G Heinrich F Garantziotis P. Targeting CD38 with daratumumab in refractory systemic lupus erythematosus. N Engl J Med (2020) 383(12):1149–55. doi: 10.1056/NEJMOA2023325 32937047

[13] Pleguezuelo DE Díaz-Simó NR Cabrera-Marante O Lalueza A Paz-Artal E Lumbreras C. Case report: Resetting the humoral immune response by targeting plasma cells with daratumumab in anti-phospholipid syndrome. Front Immunol (2021) 12. doi: 10.3389/fimmu.2021.667515 PMC8072150 33912194

[14] Zand L Rajkumar SV Leung N Sethi S Ters M Fervenza FC. Safety and efficacy of daratumumab in patients with proliferative GN with monoclonal immunoglobulin deposits. J Am Soc Nephrol (2021) 32:1163–73. doi: 10.1681/ASN.2020101541 PMC8259683 33685975

[15] Sanchez L Wang Y Siegel DS Wang ML. Daratumumab: a first-in-class CD38 monoclonal antibody for the treatment of multiple myeloma. J Hematol Oncol (2016) 9(1):51. doi: 10.1186/S13045-016-0283-0 27363983 PMC4929758

[16] van de Donk NWCJ Usmani SZ. CD38 antibodies in multiple myeloma: Mechanisms of action and modes of resistance. Front Immunol (2018) 9:2134(SEP). doi: 10.3389/FIMMU.2018.02134 30294326 PMC6158369

[17] Yamamoto C Minakata D Koyama S Sekiguchi K Fukui Y Murahashi R. Daratumumab in first-line therapy is cost-effective in transplant-eligible patients with newly diagnosed myeloma. Blood (2022) 140(6):594–607. doi: 10.1182/BLOOD.2021015220 35580269 PMC9373013

[18] Offidani M Corvatta L Morè S Nappi D Martinelli G Olivieri A. Daratumumab for the management of newly diagnosed and Relapsed/Refractory multiple myeloma: Current and emerging treatments. Front Oncol (2020) 10:624661. doi: 10.3389/FONC.2020.624661 33680948 PMC7928404

[19] Chari A Rodriguez-Otero P McCarthy H Suzuki K Hungria V Sureda Balari A. Subcutaneous daratumumab plus standard treatment regimens in patients with multiple myeloma across lines of therapy (PLEIADES): an open-label phase II study. Br J Haematol (2021) 192(5):869–78. doi: 10.1111/BJH.16980 33216361

[20] Phou S Costello C Kopko PM Allen ES. Optimizing transfusion management of multiple myeloma patients receiving daratumumab-based regimens. Transfusion (Paris) (2021) 61(7):2054–63. doi: 10.1111/TRF.16425 33960433

[21] Izaguirre EC del Mar Luis-Hidalgo M González LL Castaño CA. New method for overcoming the interference produced by anti-CD38 monoclonal antibodies in compatibility testing. Blood Transfus (2020) 18(4):290–4. doi: 10.2450/2020.0004-20 PMC7375880 32530397

[22] Tauscher C Moldenhauer S Bryant S DiGuardo M Jacob EK. Antibody incidence and red blood cell transfusions in patients on daratumumab. Transfusion (Paris) (2021) 61(12):3468–72. doi: 10.1111/TRF.16687 34617617

[23] Pirenne F Floch A Habibi A. How to avoid the problem of erythrocyte alloimmunization in sickle cell disease. Hematol Am Soc Hematol Educ Program (2021) 2021(1):689–95. doi: 10.1182/HEMATOLOGY.2021000306 PMC8877235 34889373

[24] Nickel RS Flegel WA Adams SD Hendrickson JE Liang H Tisdale JF. The impact of pre-existing HLA and red blood cell antibodies on transfusion support and engraftment in sickle cell disease after nonmyeloablative hematopoietic stem cell transplantation from HLA-matched sibling donors: A prospective, single-center, observational study. EClinicalMedicine (2020) 24. doi: 10.1016/J.ECLINM.2020.100432 PMC7327930 32637902

[25] Cutler C Kim HT Sun L Sese D Glotzbecker B Armand P. Donor-specific anti-HLA antibodies predict outcome in double umbilical cord blood transplantation. Blood (2011) 118(25):6691–7. doi: 10.1182/BLOOD-2011-05-355263 PMC3976219 21940825

[26] Spellman S Bray R Rosen-Bronson S Haagenson M Klein J Flesch S. The detection of donor-directed, HLA-specific alloantibodies in recipients of unrelated hematopoietic cell transplantation is predictive of graft failure. Blood (2010) 115(13):2704–8. doi: 10.1182/BLOOD-2009-09-244525 PMC2852369 20089963

[27] Barcellini W Fattizzo B Zaninoni A. Management of refractory autoimmune hemolytic anemia after allogeneic hematopoietic stem cell transplantation: current perspectives. J Blood Med (2019) 10:265–78. doi: 10.2147/JBM.S190327 PMC6690850 31496855

[28] Khandelwal P Teusink-Cross A Kumar AR Bleesing JJ Mehta PA Jordan MB. Daratumumab for the management of autoimmune cytopenias in children and young adults: a case series. Br J Haematol (2021) 194(5):e84–9. doi: 10.1111/BJH.17565 34046889

[29] Epperly R Santiago T Morin CE Patton K Deyo J Eshun J. Targeting plasma cells with daratumumab aids in the treatment of post-transplant autoimmune-like hepatitis. Pediatr Blood Cancer (2021) 68(11):e29290. doi: 10.1002/PBC.29290 34390168

[30] Leffell MS Jones RJ Gladstone DE. Donor HLA-specific abs: to BMT or not to BMT? Bone Marrow Transplant (2015) 50(6):751–8. doi: 10.1038/BMT.2014.331 PMC4634885 25706884

[31] Ansari M Uppugunduri CRS Ferrari-Lacraz S Bittencourt H Gumy-Pause F Chalandon Y. The clinical relevance of pre-formed anti-HLA and anti-MICA antibodies after cord blood transplantation in children. PloS One (2013) 8(8):e72141. doi: 10.1371/JOURNAL.PONE.0072141 23977232 PMC3747133

[32] Brunstein CG Noreen H DeFor TE Maurer D Miller JS Wagner JE. Anti-HLA antibodies in double umbilical cord blood transplantation. Biol Blood Marrow Transplant (2011) 17(11):1704–8. doi: 10.1016/J.BBMT.2011.04.013 PMC3168972 21601639

[33] Takanashi M Atsuta Y Fujiwara K Kodo H Kai S Sato H. The impact of anti-HLA antibodies on unrelated cord blood transplantations. Blood (2010) 116(15):2839–46. doi: 10.1182/BLOOD-2009-10-249219 20628152

[34] Spica D Junker T Dickenmann M Schaub S Steiger J Rufli T. Daratumumab for treatment of antibody-mediated rejection after ABO-incompatible kidney transplantation. Case Rep Nephrol Dial (2019) 9(3):149–57. doi: 10.1159/000503951 PMC6902247 31828078

[35] Dossier C Prim B Moreau C Kwon T Maisin A Nathanson S. A global antiB cell strategy combining obinutuzumab and daratumumab in severe pediatric nephrotic syndrome. Pediatr Nephrol (2021) 36(5):1175–82. doi: 10.1007/S00467-020-04811-0 PMC7594934 33118048

[36] Cerrano M Bonifacio M Olivi M Curti A Malagola M Dargenio M. Daratumumab with or without chemotherapy in relapsed and refractory acute lymphoblastic leukemia. a retrospective observational campus ALL study. Haematologica (2022) 107(4):996–9. doi: 10.3324/HAEMATOL.2021.279851 PMC8968887 35021604

[37] Cottini F Huang Y Williams N Bumma N Khan AM Chaudhry M. Real world experience of daratumumab: Evaluating lymphopenia and adverse events in multiple myeloma patients. Front Oncol (2021) 10:575168. doi: 10.3389/FONC.2020.575168 33659205 PMC7917249

[38] Paul Y Aguirre LE Basher F Miao F Koru-Sengul T Hoffman JE. Hypogammaglobulinemia and its implications in patients treated with daratumumab: A single institution experience. Blood (2019) 134(Supplement_1):3131. doi: 10.1182/BLOOD-2019-127247

