# Supplementary material for: Case report: Daratumumab treatment in pre-transplant alloimmunization and severe hemolytic anemia
Source: Front Immunol. 2022 Nov 29;13:1055473. doi: 10.3389/fimmu.2022.1055473 (PMC9744936; doi:10.3389/fimmu.2022.1055473)
Supplement: Supplementary file 1 [file DataSheet_1.pdf]

Supplementary Material

Table A. Serum antibody profile for patient 1

	Before Daratumumab treatment	10 days after treatment	1 months after treatment	2 months after treatment	3 months after treatment
**Ab-e**	positive	positive	positive	positive	negative
**Anti-E**	positive	negative	negative	negative	negative
**Anti-Fy**	positive	negative	negative	negative	negative
**Anti-C**	positive	positive	positive	negative	negative
**Anti-D**	positive	positive	positive	negative	negative

Table B: Post second HSCT chimerism for patient 2

Days from transplant		Complex post-transplant chimerism	CD33 chimersim	CD3 chimersim
T+30	Donor	98%	97%	48%
	Recipient	2%	3%	52%
T+60	Donor	94%	97%	5%
	Recipient	6%	3%	95%
T+120	Donor	76%	97%	6%
	Recipient	24%	3%	94%
T+365	Donor	89%	100%	54%
	Recipient	11%	0%	46%
T+3 years	Donor	86%	100%	63%
	Recipient	14%	0%	37%

Table C. Platelet reactive antibodies for patient 3

Date	Class I	Class II
June 2021	Multiple antibodies	Multiple antibodies<br><br>Weak DR0, DQ7, DQA03:02
August 2021	Multiple antibodies<br><br>A1, A36, B8, B18, B35, B38, B39, B41, B48, B49, B50, B51, B53, B59, B61, B62, B64, B67, B71, B72, B75, B77, B78, B40:05	Negative
September 2021	Multiple antibodies<br><br>A1, A36, B35, B39, B51, B53, B54, B71, B75, B78, weak B77	Negative
November 2021	Weak B35 antibody	Negative
